# Supporting adjuvant endocrine therapy adherence in women with breast cancer: the development of a complex behavioural intervention using Intervention Mapping guided by the Multiphase Optimisation Strategy

**DOI:** 10.1186/s12913-022-08243-4

**Published:** 2022-08-24

**Authors:** Sophie M. C. Green, David P. French, Christopher D. Graham, Louise H. Hall, Nikki Rousseau, Robbie Foy, Jane Clark, Catherine Parbutt, Erin Raine, Benjamin Gardner, Galina Velikova, Sally J. L. Moore, Jacqueline Buxton, ROSETA investigators, Michelle Collinson, Rachel Ellison, Hollie Wilkes, Suzanne Hartley, Ellen Mason, Amanda Farrin, Rebecca Walwyn, Jo Waller, Daniel Howdon, Jamie Metherell, Samuel G. Smith

**Affiliations:** 1 Leeds Institute of Health Sciences, University of Leeds, Clarendon Way, Leeds, LS2 9NL UK; 2 Manchester Centre for Health Psychology, University of Manchester, Manchester, UK; 3 Department of Psychology, Queen’s University Belfast, Belfast, UK; 4 Leeds Institute of Clinical Trials Research, University of Leeds, Leeds, UK; 5 St. James’s University Hospital, Leeds Teaching Hospitals NHS Trust, Leeds, UK; 6 Department of Psychology, Institute of Psychiatry, Psychology and Neuroscience, King’s College London, London, UK; 7 Leeds Institute of Medical Research at St James’s, University of Leeds, St James’s University Hospital, Leeds, UK; 8 Leeds Cancer Centre, Leeds Teaching Hospitals NHS Trust, St James’s University Hospital, Leeds, UK

**Keywords:** Breast cancer, Medication adherence, Intervention mapping, Multiphase optimisation strategy

## Abstract

**Background:**

Adjuvant endocrine therapy (AET) reduces the risk of breast cancer recurrence and mortality. However, up to three-quarters of women with breast cancer do not take AET as prescribed. Existing interventions to support adherence to AET have largely been unsuccessful, and have not focused on the most salient barriers to adherence. This paper describes the process of developing four theory-based intervention components to support adherence to AET. Our aim is to provide an exemplar of intervention development using Intervention Mapping (IM) with guidance from the Multiphase Optimisation Strategy (MOST).

**Methods:**

Iterative development followed the six-stage IM framework with stakeholder involvement. Stage 1 involved a literature review of barriers to adherence and existing interventions, which informed the intervention objectives outlined in Stage 2. Stage 3 identified relevant theoretical considerations and practical strategies for supporting adherence. Stage 4 used information from Stages 1-3 to develop the intervention components. Stages 1-4 informed a conceptual model for the intervention package. Stages 5 and 6 detailed implementation considerations and evaluation plans for the intervention package, respectively.

**Results:**

The final intervention package comprised four individual intervention components: Short Message Service to encourage habitual behaviours surrounding medication taking; an information leaflet to target unhelpful beliefs about AET; remotely delivered Acceptance and Commitment Therapy-based guided self-help to reduce psychological distress; and a website to support self-management of AET side-effects. Considerations for implementation within the NHS, including cost, timing and mode of delivery were outlined, with explanation as to how using MOST can aid this. We detail our plans for the final stage of IM which involve feasibility testing. This involved planning an external exploratory pilot trial using a 2^4-1^ fractional factorial design, and a process evaluation to assess acceptability and fidelity of intervention components.

**Conclusions:**

We have described a systematic and logical approach for developing a theoretically informed intervention package to support medication adherence in women with breast cancer using AET. Further research to optimise the intervention package, guided by MOST, has the potential to lead to more effective, efficient and scalable interventions.

**Supplementary Information:**

The online version contains supplementary material available at 10.1186/s12913-022-08243-4.

## Background

Breast cancer is the most common cause of cancer death in women [1]. Around 75% of breast cancers are oestrogen receptor-positive (ER+) [2]. Adjuvant endocrine therapy (AET), including tamoxifen and aromatase inhibitors (AIs; anastrozole, letrozole, exemestane) are prescribed to women with ER+ breast cancer to reduce the risk of cancer recurrence and mortality [3, 4]. AET is prescribed for 5-10 years [5], with 7-8 years potentially the optimal duration [6–9]. However, up to three-quarters of patients do not take AET as prescribed [10–13]. Non-adherence and non-persistence (not continuing to take the medication for the prescribed duration) are linked to an increased risk of recurrence, lower survival and reduced quality-adjusted life years [14–16]. Improving adherence to AET could reduce healthcare costs associated with cancer recurrence [15].

Modifiable barriers to AET adherence have been identified [17–20]. Most existing interventions do not target multiple factors associated with adherence, and predominantly consist of solely educational interventions, such as leaflets [21–23]. Such interventions have either been ineffective or yield small effect sizes [21–23]. This is characteristic of interventions aiming to support adherence across a wide range of chronic conditions, highlighting the need for improved interventions to support adherence more generally [24]. Considerations of theory in interventions aiming to support AET adherence are often lacking, with little transparency of the intervention development process. The UK Medical Research Council Framework (MRC) for developing and evaluating complex interventions, and INDEX guidance (Identifying and assessing different approaches to developing complex interventions) suggest interventions should be developed based on theory in a systematic manner to aid replication and implementation [25–27]. Developing interventions grounded in theory can improve our understanding of why an intervention is successful or unsuccessful. Intervention mapping (IM) is a systematic approach that can be used to develop theory and evidence-based health interventions that can fulfil MRC and INDEX guidance [28]. It consists of six stages that cover designing, implementing and evaluating an intervention, and it promotes relevant stakeholder engagement throughout development [28]. IM has previously been used to develop interventions targeting adherence [29–31] and women with breast cancer [32, 33].

The AET adherence trials published to date are mostly evaluated using parallel groups randomised controlled trials (RCTs). RCTs can definitively evaluate whether an intervention package as a whole has a statistically significant effect compared with a comparator. However, RCTs alone are unable to explain which components of a complex intervention affect the outcome, whether there are interactions between intervention components, and whether the benefits of an intervention component are justified based on resource demands. The Multiphase Optimisation Strategy (MOST) addresses these limitations [34] by optimising interventions based on the performance of individual intervention components relative to resource constraints. MOST consists of three phases: (1) preparation, in which intervention components are developed; (2) optimisation, in which efficient experimental designs, which estimate main effects and interactions between intervention components, are used to build an optimal intervention package; and (3) evaluation, in which the optimised intervention package is evaluated, typically using a parallel groups RCT.

There are important factors to consider when developing interventions within the MOST framework. These include ensuring each intervention component targets a specific mediating variable, that there is minimal overlap between the content of the intervention components, and that thought is given to the challenges of delivering all intervention components within a single package [35]. Combining the IM and MOST frameworks enables these considerations of MOST to be acknowledged systematically throughout every stage of development within IM. This paper describes the development of an intervention package to support AET adherence in women with early-stage breast cancer, aiming to provide an exemplar of how to incorporate IM into the MOST framework.

## Methods

We progressed through six stages of IM in line with published guidance (Table 1) [28]. We followed the Guidance for reporting intervention development studies in health research (GUIDED) [36].

**Table 1:** Adapted Intervention mapping framework

Stage	What was done?
Stage 1- Needs assessment	• Literature review of the problem of non-adherence, barriers to adherence, and existing interventions to support adherence to AET • Population of interest described • Overall goal for the intervention established and stated
Stage 2- Intervention objectives	• Selection of behavioural determinants to be targeted, based on needs assessment and context of intervention • Intervention component objectives stated • Conceptual model created, detailing causal change pathways and hypothesised interactions between components
Stage 3- Intervention Design	• Theories relevant to each determinant identified were considered • Existing interventions explored, informed by the needs assessment and practical applications considered
Stage 4- Intervention development	• Intervention components finalised based on Stage 3 • Intervention development work completed; intervention materials created and drafted • Stakeholder input from clinicians, patients and research team used to refine intervention materials
Stage 5- Implementation planning	• Implementation in the development phase discussed, and MOST optimisation objective outlined
Stage 6- Evaluation plan	• Hypothesised interactions between intervention components outlined and explained • Evaluation plan considered

Key: *MOST* Multiphase Optimisation Strategy

### Stage 1: Needs assessment

The needs assessment involved three sub-stages: (1) a literature review to understand the extent of non-adherence in women prescribed AET; (2) a literature review to understand the barriers to AET adherence, predominantly focusing on existing reviews identified through backward citation searching [11, 18, 20, 37–45]; and (3) a rapid review and search of trial registries to identify published interventions and ongoing trials addressing AET adherence. The terms “hormone therapy” “breast cancer”, “adherence”, “intervention” and their variations were used. One author (SG) screened the texts and extracted data. The needs assessment informed the primary aims of the intervention package.

### Stage 2: Intervention objectives

Modifiable determinants of AET adherence to be targeted in the intervention package were selected based on the results of Stage 1. For each determinant chosen, specific objectives for an intervention component to target were defined. Stage 2 considered how IM could be incorporated into MOST. An important aspect of the preparation phase of MOST is the conceptual model [35], similar to the logic model produced in IM. A conceptual model details how each intervention component is expected to change the outcome. It is recommended that each intervention component targets one specific mediating variable to aid decision making within the optimisation phase [46]. The intervention components should be reasonably independent to ensure one component does not depend on the presence of another. This means that the delivery of a component, and what the participant receives, should not be affected by the levels of the other components they may receive [35]. Conceptual model development was iterative; draft illustrations of the model were created, discussed within the research team, and with Patient and Public Involvement (PPI) members.

### Stage 3: Intervention design

For each determinant of AET adherence that we identified and selected in Stages 1 and 2, existing interventions and associated literature were explored to identify suitable theories, evidence-based behaviour change methods and practical strategies that could address them. We identified psychological theories specific to the determinants, and considered how these theories could inform the development of the intervention components. The research team, in collaboration with PPI members, used this evidence to discuss which strategies were most likely to be effective and implementable within the UK healthcare system.

### Stage 4: Intervention development

Four intervention components were developed; two new components and two adapted from existing interventions. Clinician, researcher and patient views were considered throughout. To aid future replication, the intervention components were coded onto the Behaviour Change Techniques taxonomy (BCTTv1) by one author (SG) who had completed BCTTv1 training [47]. Component coding was discussed between members of the research team (SG, SS, CG, LH). Disagreements were discussed and resolved. To evaluate readability, a Flesch-Kincaid reading age and grade level was calculated for each component [48]. We aimed for a reading grade level of 7 to 8 which are described as ‘fairly easy’ and ‘standard’ levels respectively [48].

### Stage 5: Implementation planning

Implementation factors such as cost, time and delivery method were considered. An optimisation objective by which the intervention will be optimised was specified, as recommended by the MOST framework. The optimisation objective operationalises the primary outcome, and key considerations that the optimised intervention should fit within, such as effectiveness, cost and time [49].

### Stage 6: Evaluation plan

The research team selected the evaluation design, and prepared a protocol for a pilot trial (ISRCTN: 10487576). We specified expected interactions between intervention components, based on theoretical assumptions identified in Stage 3. The a priori specification of hypothesised interactions is important, as components forming the interactions will be prioritised when deciding the optimised intervention package [50].

### Patient and public involvement (PPI)

Our PPI panel of five members met remotely with two researchers (SG, ER) every 2-3 months throughout the development phase. The panel comprised five women with a diagnosis of breast cancer and experience of taking AET, recruited by advertising through a charity supporting people affected by cancer. Members were compensated for their time.

## Results

### Stage 1: Needs assessment (findings from literature reviews)

#### Extent of nonadherence

Adherence to AET is suboptimal, with up to 73% not taking it as prescribed [11, 41]. A large number of women discontinue AET within the first year [51]. Adherence diminishes over time, with up to 50% of women being non-adherent within 5 years [10, 13]. Unintentional nonadherence (e.g. forgetting to take medication) may be more prevalent than intentional nonadherence (e.g. deciding to miss a tablet) [52–54].

#### Factors associated with adherence and nonadherence

Barriers to and facilitators of AET adherence were identified (Table 2).

**Table 2:** Summary of barriers to AET adherence

Factor associated with adherence	Explanation	Evidence
Experience of side effects^a^	Barrier: Increased frequency and intensity of side effects	[11, 18, 20, 39, 42–45, 55–58]
Medication beliefs^a^	Facilitator: more beliefs about the necessity of AET Barrier: more concerns about AET	[11, 18–20, 37, 39–41, 43, 45]
Illness perceptions^a^	Facilitators: beliefs that certain lifestyle behaviours can cause a recurrence Barriers: low risk perception of recurrence, high tamoxifen consequences, belief that psychological factors cause a recurrence	[56, 57, 59]
Knowledge/ information available^a^	Barriers: Lack of knowledge of side effects and the mechanisms of AET	[39]
Psychological distress^a^	Barriers: Increased distress (including depression and anxiety)	[20, 60]
Forgetfulness^a^	Barriers: forgetting to take medication, memory difficulties	[18, 41, 61]
Social support	Facilitators: Increased social support	[11, 37, 39, 40, 42, 43, 57]
Self-efficacy	Facilitators: Increased self-efficacy	[37, 39, 43, 45]
Patient-physician communication	Facilitators: Better patient-physician relationship	[20, 37, 40, 42, 43]

Key: *AET* Adjuvant endocrine therapy

^a^Indicates factor included within the conceptual model for the intervention in Stage 2

##### Side-effects

Literature has suggested that the frequency, severity and inability to manage side-effects are common barriers to AET adherence and persistence [11, 18, 20, 39, 42–45, 62]. However, some reviews have questioned this relationship, citing inconsistent evidence [37, 42]. Qualitative studies highlight reasons for non-adherence including the impact of side-effects on quality of life [17], side-effects outweighing the benefits [17, 58], a lack of understandable information about the range and intensity of side-effects [58, 61], and women feeling unsupported in managing side-effects [17, 55, 58]. There is a clear demand for information about side-effects and their management [63].

##### Medication beliefs and illness perceptions

Necessity beliefs and concerns about AET, and the cost-benefit balance between these are associated with reduced adherence [11, 18–20, 37, 39–41, 43, 45]. For example, adherent women tend to report strong necessity beliefs, such as “Tamoxifen is keeping me alive”, AET helps them to feel in control, and that AET will enable them to stay alive for their family [17, 61]. In contrast, less adherent women report more concerns, such as AET benefits not being worth the reduced quality of life, and worry about the chance of cancer elsewhere [17]. Representations of breast cancer, such as believing the likelihood of recurrence is low, are also associated with lower adherence [56, 57].

##### Knowledge of medication

Lower knowledge about AET is associated with reduced adherence [39]. Women consistently report receiving insufficient information about AET [17, 55]. Approximately one fifth of breast cancer survivors in a Dutch survey did not know how AET worked, but wanted further information, and a third did not know how large the risk reduction effect was [53].

##### Psychological distress

Immediatley following active treatment, approximately half of women with breast cancer report higher levels of psychological distress than observed in the general population [20, 64, 65]. Psychological distress in breast cancer can include rumination and worry about breast cancer recurrence, difficulties in returning to ‘normal’, and distress from AET side effects [17, 58, 63]. Higher levels of distress are associated with lower adherence [20, 60], although some inconsistencies with this relationship have been observed [42, 66].

##### Forgetfulness

Women with breast cancer commonly report memory problems following chemotherapy, which can increase forgetfulness and consequently unintentional nonadherence [18, 37, 41, 61, 67–69].

##### Additional barriers to AET adherence

Social support, patient-physician communication and self-efficacy have also been identified as barriers to AET adherence [11, 20, 37, 39, 40, 42, 43, 57, 70]. Women often feel abandoned when ending active treatment and being discharged from care [71]. Higher social support from family, friends and other breast cancer survivors are associated with improved adherence and persistence [11, 37, 39, 40, 42, 43, 57, 70]. Self-efficacy in the patient-physician interaction (confidence in the ability to get medical information from a physician [39, 43, 72]), and perceived self-efficacy in relation to learning about and taking AET [37, 39, 43] are associated with higher adherence [37, 39, 43]. Patient-reported positive relationships with physicians are associated with higher adherence [20, 37, 40, 42, 43], specifically, the quality and person-centeredness of the relationship, frequency of communication, and sufficiency of information received about AET [43].

#### Existing interventions supporting adherence

We identified 16 published trials evaluating interventions targeting adherence to AET (Table 3) and 15 ongoing trials (Additional file 1). Within the 16 published trials, there was little high-quality evidence that these interventions were effective. Of the 16 published interventions, six reported statistically significant improvement in adherence. Two of those with significant findings were pilot trials and therefore were not designed to examine efficacy, two found significant findings in post-hoc analyses, and for one, a significant effect was not maintained at follow up. Six published trials evaluated interventions composed only of educational materials which were not effective in supporting adherence [73–78]. The theoretical basis and development process were inadequately described for most published interventions.

**Table 3:** Existing interventions supporting adherence to AET in women with breast cancer

Authors	Description of Intervention	Intervention modality	AET type	Design	Key results (adherence related outcomes)	Theory that informed the intervention
Ell et al. (2009) [73]	Written information plus structured ‘patient navigation’ phone interviews consisting of education, addressing barriers to adherence, problem solving, self-management support and emotional support.	Written information and telephone	All	2 arm RCT- enhanced usual care (information) vs written information plus patient navigation	No significant difference; 67% vs 69% (*p* = 0.80).	Health Belief model and socio-cultural explanatory theory
Yu et al. (2012) [74]	PACT materials used. Patient education (welcome pack and quarterly newsletters) with information about breast cancer and adherence. Follow up reminder calls.	Written information and telephone	Anastrozole or letrozole	Prospective, multicentre controlled observational study	No significant difference; 95.9% vs 95.8% one-year persistence rate (*p* = 0.95).	None reported
Ziller et al. (2013) [75]	COMPAS study. Letter group: 8 personalized motivational reminder letters were sent over 2 years with information on topics side effects and treatment. A breast cancer information leaflet containing information on topics such as nutrition and sport. Reminder phone calls: 8 telephone calls over 2 years which used motivational interviewing to address any questions, challenges to adherence, provide information and reminders.	Written information/ telephone	AI	3 arm RCT- usual care vs letters vs telephone calls	No significant difference in adherence in primary analysis. In post hoc analysis when pooling the intervention arms, adherence increased significantly in the intervention arms vs control (*p* = 0.039).	Learning theory
Hadji et al. (2013) [76]	PACT Program: educational materials sent to participants (9 mailed letters and brochures), monthly reminders on persistence to endocrine therapy, gift items sent e.g. 7 day tablet box, pocket mirror. Educational materials included information on relevant issues such as side effects, efficacy, nutrition, communication.	Written information	Anastrozole	RCT- usual care vs written information	No significant difference in compliance at 12 months (*p* = 0.81).	None mentioned
Neven et al. (2014) [77]	CARIATIDE program. PACT materials used- welcome pack and 9 letters and brochures mailed out, containing information on side effects, exercise, diet, communication.	Written information	AI	Randomized, parallel group observational study; usual care vs intervention	No significant difference in compliance between arms at 12 months (*p* = 0.4524). In Finland/Sweden, compliance was significantly higher in the intervention arm (*p* = 0.0246).	None mentioned
Graetz et al. (2018) [79]	App: Web based app in which participants asked to record symptoms and report adherence in the past 7 days. Alerts sent to care team for any concerns. App+ reminder: Web based app in which participants asked to record symptoms and report adherence in the past 7 days. Alerts sent to care team for any concerns. Weekly reminders sent to use the app via text or email.	App and text or email	AI	Pilot RCT- app use only vs app use plus reminders to use app	Proportion of patients adherent in the experimental group (100%) was greater than control group (72.7%); *p* < 0.05.	None mentioned
Heisig et al. (2015) [80]	Enhanced information leaflet and 15-minute phone calls sessions including information on the mechanisms of AET, benefits and side effects.	Written information and telephone	Any	Interventional single cohort study	Greater adherence observed at 3-month follow-up.	None mentioned
Markopoulas et al. (2015) [78]	PACT materials. Educational materials sent to participants 9 times in 1 year, consisting of information on side effects, communication, sport, nutrition, benefits, tips on how to take AET.	Written information	Anastrozole or letrozole	RCT- standard care vs intervention	No significant difference in compliance or persistence between the groups at 12 months.	None mentioned
Castaldi et al. (2017) [81]	Patient navigation program. Initial visit include assessment of barriers to adherence. Navigator provides reminder calls prior to follow up appointments, meets patients at outpatient appointments and on day of surgery, and a financial consultation where required.	Patient navigation	Tamoxifen and AI	Non randomized, historical care vs navigated care	68.6% adherence in standard care vs 100% in patient navigation (*p* < 0.0001).	None mentioned
Hershman et al. (2020) [82]	SMS messages sent twice weekly over 36 months. Content included overcoming barriers to medication adherence, cues to action, statements related to medication efficacy and reinforcements of the recommendation to take the medication. 40 messages repeated over intervention.	Text messaging	AI	RCT; text messages vs no text messages	No significant difference between text messages (55.55%) and no text messages (55.4%) at 36 months.	None mentioned
Moon et al. (2019) [33, 83]	Self-directed paper booklet designed in line with CBT and behaviour change theory. Included sections to modify beliefs about recurrence and the medication, to help manage side effects and to increase perceived behavioural control.	Written information	Tamoxifen	Pilot trial; no control group	Primary outcomes were feasibility and retention. Change from 100 to 91% who were non adherent after intervention. D = 0.31 for improvement of unintentionally non adherent women.	Common sense model and theory of planned behaviour
Bhandari et al. (2019) [84]	Prescriptions given in a 30-day bubble pack with labelled day of the week; dispensed as 1- or 3-month supply.	Medication packaging	Tamoxifen and AI’s	Single arm prospective investigational pilot study	Suggestion of improved adherence with bubble packaging (no control arm)	None mentioned
Tan et al. (2020) [85]	Weekly SMS reminders sent on a Monday morning reading “Mdm <NAME> please be reminded to take your anti-cancer medicine as instructed by your doctor. Take one tablet once every day.”.	Text messaging	All	Open level, multi centre prospective RCT	Higher percentage of adherence in SMS (72.4%) vs standard care (59.5%) at 6 months (*p* = 0.034), but not at 1 year (*p* = 0.617). No difference in serum hormone levels.	None mentioned
Krok-Schoen et al. (2019) [86]	Daily text message reminders focusing on initiation, continuation and adherence to prescribed dose; 14 messages repeated. Dynamic intervention in which participants complete weekly surveys on an app. Participants received feedback based on survey responses; either encouraging messages or problem solving. Physicians notified and patient has option to leave voice message and share with physician.	Text messaging and app	Tamoxifen or AI	Pilot trial; no control group	Significant improvement for self-reported medication adherence (*p* = 0.015), significant decreases in oestradiol, oestrogen and estrone hormone levels (*p* < 0.001).	None mentioned
Labonte et al. (2020) [32]	Community based pharmacy intervention; motivational interviewing given by pharmacists in brief individual consultations. Discussions focused on mode of action of AET, side effect coping and benefits of the medication.	In person (pharmacist)	All	Intervention mapping development	N/A- development paper	Theory of planned behaviour, motivational interviewing
Getachew et al. (2018) [87]	Breast care nurses were trained as navigators to improve patient adherence in rural Ethiopia	Breast nurse navigators	Tamoxifen	RCT	N/A- protocol abstract only	None mentioned

Key: *RCT* Randomised Control Trial, *PACT* Patients Anastrozole Compliance to Therapy, *COMPAS* Compliance in Adjuvant treatment of primary breast cancer Study, *CARIATIDE* Compliance of Aromatase Inhibitors Assessment in daily practice through educational approach, *AET* Adjuvant endocrine therapy, *SMS* Short messaging service, *CBT* Cognitive behavioural therapy, *AI* Aromatase inhibitor

#### Intervention goals

The needs assessment established the overall goal of the programme; to develop a multi-component intervention to improve AET adherence in women with early-stage breast cancer. This will be determined using primary outcome data within the optimisation phase. All barriers to AET adherence identified in Stage 1 were considered in Stage 2.

### Stage 2: Intervention objectives

Based on findings from Stage 1, and following discussion within the research team and agreement from patient representatives, four main intervention targets were selected; living with side effects, medication and illness beliefs, forgetfulness and psychological distress. These cover a range of intentional and unintentional barriers to adherence. Table 4 summarises identified determinants and the specific intervention component objectives. Illness perceptions and knowledge can affect medication beliefs through providing an understanding of how the medication works, which can enhance beliefs about its necessity [88, 89]. We therefore targeted knowledge in combination with medication beliefs.

**Table 4:** Summary of intervention components to target determinants

Determinant	Intervention component objective	Strategy	Intervention component	Description of intervention component	BCT’s targeted	Theoretical Basis
Management of side effects	Increase ability to self-manage side effects Reduce impact of side effects	Inform patients of self-management strategies for common side effects	Self-management website	A website for self-management of side effects. Strategies to manage side effects with a summary of the strength of evidence for that side effect in a patient-friendly manner. Side effects included are arthralgia, fatigue, vulvovaginal symptoms, gastrointestinal symptoms, hot flushes and sleep difficulties.	1.2, 3.1, 3.3, 4.1, 5.1, 5.3, 5.6, 6.2, 6.3, 9.1, 11.1, 12.2, 12.5, 12.6	
Medication and illness beliefs	Increase beliefs about the necessity of using AET beliefs	Provide information on how AET works and the benefits of AET.	Information Leaflet	A written information leaflet with five different elements: (1) An explanation of how AET works, including medical diagrams (2) Information and infographics about the benefits of AET (3) Information about the prevalence of side effects from AET (4) Answers to common concerns about AET (5) Quotes from breast cancer survivors about their experiences taking AET, and a statement highlighting that the leaflet was co-designed	1.2, 4.1, 4.3, 5.1, 5.2, 5.6, 6.2, 6.3, 9.1, 9.2, 11.2, 13.2	Necessity Concerns Framework, Common Sense Model of Illness Representations
	Reduce concerns about AET	Provide information on the prevalence of side effects, answer common concerns about AET.				
	Support formation of accurate illness perceptions	Provide information on the mechanism of AET and the benefits of AET to enhance coherence, personal and treatment control				
Knowledge	Learn about AET, including how it works, the benefits and side effects of it	Provide information about AET, it’s mechanism of action, benefits and side effect information	Information Leaflet	As above	As above	As above
Forgetfulness	Learn strategies to remember to take AET	Support the habit formation of daily medication taking and associated activities such as ordering and collecting prescriptions	SMS messages	SMS messages providing practical strategies to support taking medication regularly each day. Messages are sent in the following frequency: • 2 weeks of daily messages • 8 weeks of twice weekly messages • 6 weeks of weekly messages	1.2, 1.4^a^, 2.3^a^, 7.1^a^, 7.3, 8.3^a^, 11.3, 12.1)^a^, 12.5)^a^	Habit Theory
Psychological distress	Reduce psychological distress	Increase psychological flexibility	ACT	A guided-self help intervention based on ACT principles involving four skills: (1) Mindfulness: broad awareness of the here-and-now. (2) Unhooking: engaging and disengaging from thoughts as suits your purpose, and letting go of struggles with yourself. (3) Follow your values: ongoing engagement with your values; consistently choosing to move in meaningful directions. (4) Living beyond labels: Taking a perspective beyond labels and responding to yourself in ways that help you grown and learn The modules contain home practice tasks and are supported by individual sessions with a psychologist in the following format: (1) 15 minute introduction (2) 3 × 25 minute sessions following modules 1, 2 and 3 (3) 15 minute closing session following module 4	1.1, 1.2, 1.5, 1.6^b^, 1.7, 2.3, 2.4, 3.1^c^, 4.1, 4.4, 5.2, 5.4, 5.6, 6.1, 6.2, 8.1, 8.2, 8.7, 9.1, 9.2, 10.9, 11.3, 11.4, 13.4, 15.2, 15.3	ACT (based on relational frame theory)

1.1 Goal setting (behavior); 1.2 Problem solving; 1.4 Action Planning; 1.5 Review behavior goals; 1.6 Discrepancy between current behavior and goal; 1.7 Review outcome goal(s); 2.3 Self-monitoring of behavior; 2.4 Self-monitoring of outcome(s) of behavior; 3.1 Social support (unspecified); 3.3 Social support (emotional); 4.1 Instruction on how to perform a behavior; 4.3 Re-attribution; 4.4 Behavioral Experiments; 5.1 Information about health consequences; 5.2 Salience of Consequences; 5.3 Information about social and environmental consequences; 5.4 Monitoring of emotional consequences; 5.6 Information about emotional consequences; 6.1 Demonstration of the behavior; 6.2 Social comparison; 6.3 Information about others’ approval; 7.1 Prompts/cues; 7.3 Reduce prompts/cues; 8.1 Behavioral practice/ rehearsal; 8.2 Behavior substitution; 8.3 Habit Formation; 8.7; Graded tasks; 9.1 Credible source; 9.2 Pros and Cons; 10.9 Self-reward; 11.1 Pharmacological support; 11.2 Reduce negative emotions; 11.3 Conserving mental resources; 11.4 Paradoxical Instructions; 12.1 Restructuring the physical environment; 12.2 Restructuring the social environment; 12.5 Adding objects to the environment; 12.6 Body changes; 13.2 Framing/ reframing; 13.4 Valued self-identity; 15.2 Mental rehearsal of successful performance; 15.3 Focus on past success

Key: *BCT* Behavior change technique, *AET* Adjuvant endocrine therapy, *SMS* Short messaging service, *ACT* Acceptance and commitment therapy

^a^Refers to the BCT's selected for messages to be based on during a 1 day workshop with behavior change experts

^b^Note: Goals may be conceptualized differently in ACT (i.e. based on values) to how they are conceptualized in this taxonomy

^c^Note: The definition of this BCT states “advise on, arrange or provide social support OR non-contingent praise or reward for performance of the behaviour. It includes encouragement and counselling”. The coding of this BCT reflects the encouragement provided as part of the support sessions. It does not reflect ‘non-contingent praise or reward for performance of the behaviour’, which is not consistent with an ACT approach

Three determinants were not chosen as mediating variables within the conceptual model: social support; self-efficacy; and patient-physician communication. These factors are likely to be addressed by the intervention components already chosen. For example, support from a psychological therapist as part of one of the proposed components has the potential to reduce feelings of abandonment, thus targeting one aspect of social support. In a similar vein, providing information about AET as part of another component is likely to address barriers associated with patient-physician communication in which women report not receiving sufficient information about AET [43].

**Fig. 1:**
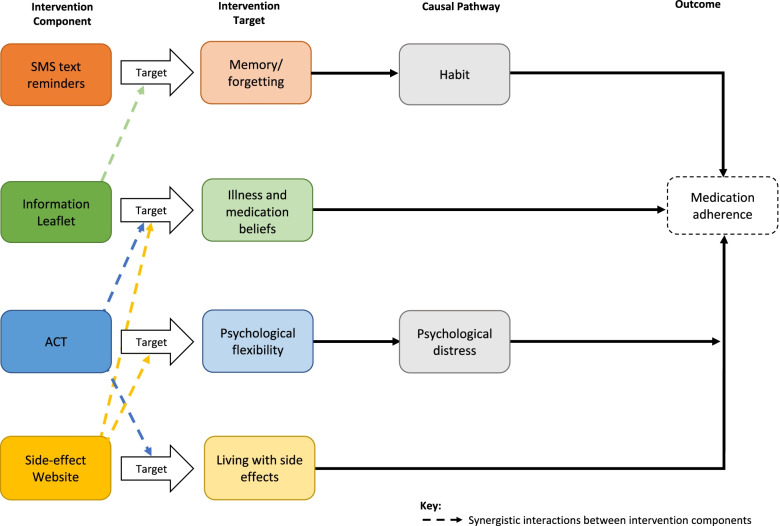
Conceptual Model

The selection of determinants based on the needs assessment, informed the conceptual model. A conceptual model, as recommended by the MRC framework, can provide a visual representation of the theoretical basis of the intervention and can improve generalisability and replicability of the intervention [26]. The development of a conceptual model is a key part of the preparation phase of MOST, in which separate intervention component targets are specified [35]. Stages 1 and 2 of IM informed the intervention target, pathway and outcome aspects of the model (Fig. 1). Stages 3 and 4 of IM provide detail on the individual intervention components. For two determinants (forgetfulness and psychological distress), there are additional stages in the conceptual model to demonstrate the pathway to adherence, described in detail in Stage 3.

### Stage 3: Intervention design

To develop intervention components according to the conceptual model, it is recommended that there is minimal overlap between the content of each intervention component to aid interpretation within the optimisation phase [35, 46]. This was considered in Stages 3 and 4. Taking the four main intervention component targets in Stage 2 (memory, illness and medication beliefs, psychological distress, side-effects), Stage 3 focused on identifying theory-based change methods and practical strategies to target these mediators.

#### Forgetfulness

Habit theory was considered to address forgetfulness, as if medication taking becomes habitual and less reliant on memory, unintentional nonadherence may reduce [90–94]. Habit theory stipulates there are multiple conceptual phases in forming a habit; deciding to act, acting on that decision, and doing so repeatedly in a manner conducive to development of behaviour cue associations [91, 94, 95]. The formation of cue-behaviour associations, as is essential to habit formation, has the potential to lead to sustained behaviour change. Habit based interventions have been successful in improving adherence in other long-term conditions [96–98]. Based on published guidance, we selected six behaviour change techniques (BCTs) related to habit theory that were feasible to target [94, 99–101] (Table 4).

Mobile messaging interventions are increasingly used to promote adherence to medications, and could be cost-effective for promoting habit formation [102–104]. Meta-analyses and systematic reviews have highlighted the significant positive effects SMS interventions could have upon medication adherence in long-term conditions, although none included women with breast cancer [102, 105]. Individual studies of SMS interventions to promote adherence by women with breast cancer have shown mixed results [82, 85, 86]. These interventions did not target habit formation specifically, and often repeated the same messages, which could cause response fatigue [102, 103, 106].

#### Medication and illness beliefs

Information provision can support the formation of medication beliefs [107, 108]. The Necessity-Concerns framework suggests patients weigh up the benefits and costs when considering a medication [109]. An extended version of the commonsense model of illness representations (CSM) highlights that cognitive and emotional illness representations, in addition to medication beliefs, influence adherence [110]. The CSM has previously been applied to the development of an intervention to support AET adherence [33]. Illness representations have been correlated with necessity and concern beliefs in women with AET [59], suggesting they could be targeted together. Providing positively framed and accurate written information about the benefits and risks of AET could increase necessity beliefs and reduce unhelpful concerns and illness representations [88, 89, 108, 111–113].

#### Psychological distress

Within a range of long-term conditions including cancer, Acceptance and Commitment Therapy (ACT) can reduce psychological distress [114, 115] and improve functioning and quality of life [114–120]. ACT is a newer type of cognitive behavioural therapy, derived from the philosophy of ‘Functional Contextualism’ and relational frame theory [121]. Consequently, ACT aims to help people engage in activity they find enriching and meaningful, even in objectively difficult situations (for example being diagnosed with cancer), by engendering a quality called psychological flexibility [121]. Psychological flexibility involves individuals approaching experiences with openness and awareness to engage more fully with their own overarching goals and values [121]. Psychological inflexibility is associated with psychological distress in breast cancer survivors [122].

Preliminary studies show psychological flexibility is positively correlated with treatment uptake and adherence in long term conditions, and that ACT could be helpful for improving medication adherence [114, 123–126]. ACT could improve overall wellbeing and reduce psychological distress by enabling individuals to function effectively alongside common emotional experiences that occur in this population [71].

#### Living with side-effects

Many side-effects women experience while taking AET can be managed without speaking to a healthcare professional [127]. Many women taking AET already self-manage their symptoms, and most want more support to do this [128]. In previous co-development work, patient representatives and healthcare professionals suggested that a website would allow patients to access side-effect management resources when required [71]. Demand for an online resource detailing evidence-based solutions to manage side-effects has also been reported elsewhere [129]. Therefore, a practical strategy to inform women about side-effects and their management was required.

As a result of Stage 3, the practical strategies to target each determinant were confirmed, to be developed in Stage 4.

### Stage 4: Intervention development

Four intervention components were developed using distinct formats: SMS messages, an information leaflet, ACT sessions, and a side-effect management website (Additional file 2). The SMS messages and information leaflet were newly developed, while the ACT sessions and side-effect management website were adapted from existing interventions [71, 130, 131]. To develop components according to the conceptual model, the same considerations were applied here as in Stage 3, to minimise duplication of information across components [35]. As a result, the four intervention components largely targeted a range of separate BCTs, with some minimal overlap (Additional file 3, Table 4). Readability of the components ranged between 11 and 14 years old (Table 5). The 12-item ‘Template for Intervention Description and Replication’ (TIDieR) checklist describes the intervention components [132] (Additional file 4).

**Table 5:** Readability of intervention components

Intervention Component	Flesch-Kincaid Grade	Age range
SMS messages	7.6	12-13 years old
Information leaflet	7.1	12-13 years old
ACT participant manuals		
Module 1	6.1	11-12 years old
Module 2	6.9	11-12 years old
Module 3	7.8	12-13 years old
Module 4	8.3	13-14 years old
Website	7.2	12-13 years old

Key: *SMS* Short messaging service, *ACT* Acceptance and commitment therapy

#### SMS development

SMS messages were co-developed using an established method for producing acceptable messages with high fidelity to the intended BCT [133]. This method has previously produced SMS messages that maintained acceptability and fidelity to intended BCTs when sent within a feasibility trial [134], and were successful in changing hypothesised mediating variables [135]. For our intervention component, behaviour change experts created messages based on BCTs during a one-day workshop, and rated the BCTs on relevance to adherence and the fidelity of individual messages to the BCT they intended to target, on a 10-point scale. Messages scoring below an a priori threshold of 5.5 were removed. The remaining messages were revised following a focus group with PPI members, and rated on acceptability by breast cancer survivors in an online survey on a 5-point Likert scale. Messages scoring below an a priori threshold of 3 were removed. An additional group of behaviour change experts rated message fidelity to the BCT on a 10-point scale, and messages scoring below an a priori threshold of 5.5 were removed [136].

The SMS intervention component will begin with 2 weeks of daily messages, as habit formation occurs most rapidly within the first 2 weeks [95, 137]. The messages will reduce to twice weekly for 8 weeks to ensure they do not become intrusive. One of the main reasons for nonadherence in an SMS trial was cited as forgetting at weekends due to a change of routine [85, 138]. Messages sent twice weekly could support medication taking in the change of routine at weekends [139]. The SMS messages will then reduce to weekly reminders for 6 weeks, as medication taking should become sufficiently habitual to persist despite a reduction in support. Frequent messages over a long period could lead to response fatigue; weekly messages are less susceptible to this effect [102, 103, 106]. It is important to reduce the frequency so that habit formation is not dependent on reminders, but is due to creating cues for medication taking [99]. To target all phases of habit formation concurrently, a combination of BCTs will be targeted throughout [94].

#### Information leaflet development

The development of the information leaflet was an iterative process. It contains five elements (Table 4). PPI members were involved throughout, including planning the content, critiquing drafts, and confirming the content of the final version. Content was informed by information from reputable sources (e.g. NHS website, MacMillan and Cancer research UK). A professional design company was commissioned to create the leaflet. Design decisions, including font size, colour contrasts and layout were informed by the Medicines and Healthcare products Regulatory Agency (MHRA) best practice for information design [140]. The leaflet underwent further refinement via patient feedback within PPI meetings, and clinical input from a consultant pharmacist.

#### Acceptance and commitment therapy (ACT) development

The ACT component was developed from an existing guided self-help intervention for improving quality of life and distress in people with muscle disorders [130, 131]. The programme, which includes common ACT techniques [141], was adapted to be relevant to women with breast cancer taking AET. It was adapted by two clinical psychologists (CG and JC) with experience in ACT and breast cancer, in collaboration with members of the research team (SS and SG). PPI members provided feedback at the planning and drafting stages. The adaptation involved rewording the participant module booklets to be relevant for women taking AET, and providing additional exercises to foster self-compassion.

The resulting intervention component involves guided self-help, consisting of four distinct modules (Table 4). Module content is presented in four participant handbooks supplemented by audio files and home practice tasks, which are conceptualised to participants as enabling them to develop four specific skills related to psychological flexibility (Table 4). The four modules are supported by five individual sessions with a practitioner psychologist ranging from 15 to 25 minutes. The sessions provide a space to discuss the module content, to reflect on experience of practising the skills in everyday life, and to consider their helpfulness.

#### Website development

The side-effect management website was developed as part of an existing intervention for women taking AET [71]. The content of the website was informed by an umbrella review of self-management strategies for side-effects in AET [127] and suggestions from breast cancer survivors. Suggestions included the use of patient narratives [71], which have been shown to improve engagement [142, 143]. To adapt the intervention, design elements were changed, and some sections were removed to ensure this was a standalone component only targeting side-effects [35].

### Stage 5: Implementation planning

The optimisation objective chosen was to create the most effective intervention package achievable that costs no more than £3997 per patient. This optimisation objective was based on health economic modelling [15]. An intervention that is effective at showing an absolute improvement of 10% in adherence would be considered cost effective if it could be delivered for less than £3997 per patient. The optimisation objective will be considered in the optimisation phase to ensure the intervention package developed is likely to be within cost-effectiveness thresholds.

Discussions with stakeholders highlighted the following considerations for potential implementation and maintenance of the intervention components. The SMS, information leaflet, and website components all represent relatively low-cost components with relatively modest maintenance needs. Therapist hours, cost and mode of delivery were considered in detail for the ACT component. There was a large amount of stakeholder engagement throughout the ACT adaptation process, involving patient representatives, clinical psychologists and service managers to consider feasibility of implementation within the NHS [71]. A guided self-help intervention was chosen by the research team in collaboration with patient representatives, as it required a lower number of therapist hours to deliver. This follows a similar approach to the Improving Access to Psychological Therapies (IAPT) model, which uses brief guided self-help interventions and has been widely implemented in the NHS [144]. Remote delivery was chosen as it can benefit patients through eliminating the need to travel to sessions. Remote delivery also reduces the need to identify clinic rooms which can be a constraint in NHS psychological services. The option of telephone or videoconferencing was chosen to reduce exclusion of those without access to videoconferencing software or a private space. Guidance for how to use videoconferencing platforms will be given.

### Stage 6: Evaluation plan

#### Expected interactions between intervention components

Hypothesised synergistic interactions are displayed using dashed lines in Fig. 1 and explained below. In a synergistic interaction, the presence of one component enhances the effect of another. In such a case, the effect of two or more factors (factors refer to independent variables in a factorial experiment) is greater than would be expected based solely on the main effects of these factors [145]. No antagonistic interactions (the presence of a component reduces the effect of another) were hypothesised.

##### SMS messages and information leaflet

Habit formation consists of multiple phases [91, 94, 95]. SMS reminders will specifically target initiation, and repetition conducive to formation of cue-behaviour associations. The other phase, deciding to take the medication, relies on motivation to engage in the behaviour [94], which could be influenced by a positive necessity-concerns differential [146]. Therefore, we hypothesise the information leaflet will contribute to and enhance the process of habit formation, resulting in a greater overall effect on adherence.

##### ACT and information leaflet

Some processes in ACT will indirectly target emotional representations of illness, that are associated with medication beliefs [37]. For example, ACT-based skills that help one ‘unhook’ from distressing thoughts, could positively affect emotional representations, such as reducing fear of recurrence [147]. Reducing emotional representations such as worry may synergistically reduce concerns about AET [59]. Therefore, ACT and the information leaflet together may have a greater effect on medication adherence than each component alone.

##### Website and information leaflet

A major concern women have with AET is side-effects [17, 55, 61, 148]. From a causal learning theory perspective to adherence, bottom-up learning (where actual experiences shape beliefs) may occur in which experiences with side-effects could shape medication beliefs [107]. The website may have a positive effect on experience of side-effects, while the information leaflet may reduce concerns, leading to a more positive necessity-concerns differential [146]. Consequently, combining the website and information leaflet may have an overall greater impact on adherence.

##### ACT and website

Engagement in ACT techniques may increase willingness to tolerate side-effects when medication-taking is consistent with values, and can reduce symptom interference [116, 120, 121, 149]. Engagement in the ACT component in combination with self-management strategies from the website, may therefore increase one’s ability to live well alongside side-effects, reducing their interference with meaningful functioning, consequently leading to greater adherence.

Additionally, use of the website may reduce side-effects. If the impact of side-effects is reduced, participants may be able to focus on life-enriching activities consistent with their values [121, 126, 149]. Therefore, use of the website may enhance engagement in the ACT component, leading to a greater overall effect upon adherence.

#### Specification of plans for evaluation design

We prepared a protocol for an external exploratory pilot trial using a 2^4-1^ fractional factorial design, with a nested process evaluation, to determine the acceptability and fidelity of the intervention components, and the feasibility of evaluating them in a larger optimisation trial [46, 150]. If progression criteria are met, we will proceed to an optimisation trial using a 2^4^ factorial design. A full factorial design is likely to be needed for the optimisation trial. This is because we have specified multiple 2-way interactions in Stage 6, which would be aliased with other potentially important effects in a fractional factorial design [151].

## Discussion

We have demonstrated a transparent and systematic approach to the development of a complex behavioural intervention designed to support medication adherence in women with breast cancer. Using an iterative IM approach, and informed by the MOST framework, we used existing evidence, behavioural science theory, and patient experience to design an intervention package consisting of four intervention components (SMS, information leaflet, ACT, website) targeting key determinants of AET adherence.

Our study illustrates how intervention development can be guided by both IM and the MOST framework [34, 35, 46]. Our plans to use a factorial design to optimise the intervention package will help delineate the individual contributions and interactions between the intervention components. This optimisation process aims to develop interventions that are more effective, efficient and scalable [34, 46, 152]. This approach could accelerate knowledge in intervention development through improved understanding of which aspects of an intervention work and why [153]. Combining IM with MOST could therefore be a more efficient method to develop and evaluate interventions, than using IM alone.

The MOST framework influenced key points in the intervention development process, namely, ensuring each component targeted a specific mediator, consideration of how the intervention components fit together as a package, and ensuring each component was distinct. Using a staged approach such as IM enabled us to consider these points throughout development. To avoid the possibility of developing a disjointed intervention package we ensured continuity in the aesthestics of each component.

Targeting all barriers to adherence identified in the needs assessment was a challenge. A pragmatic decision was made not to include all barriers identified in Stage 1 in the conceptual model. Firstly, adding more intervention components increases the number of experimental conditions required in a factorial design. For example, adding three extra components would lead to a 2^7^ factorial design requiring 128 experimental conditions if using a full factorial design. This may not be feasible to deliver. If we demonstrate that it is feasible to undertake a 2^4-1^ experimental design in the proposed pilot trial, additional intervention components could be considered in the future, as fractional factorial designs can be more efficient in these circumstances. Secondly, barriers such as social support and patient-physician communication are likely to require complex designs. For example, while the ACT component does provide a degree of social support, it could be argued that this could be more adequately addressed with a group-based psychotherapy intervention. However, evaluating group-based intervention components using a factorial experiment may necessitate more complex, multilevel designs [154]. While such designs exist, they have rarely been used and methodological expertise and guidance are lacking. This issue led to uncertainty in deciding between a group-based or an individual psychotherapy component. Importantly, the conceptual model presented in this paper has not yet been tested, and can be refined in the future as further information is collected. For example, should we receive strong feedback from women receiving these interventions within the planned pilot trial that they would have preferred a group-based approach, we will give further consideration to evaluating it in a future optimisation trial. This decision will also be guided by the results of a separate pilot trial testing a group-based ACT intervention currently being undertaken by the authors (LH, SS, CG, JC) [155], alongside qualitative feedback within our planned process evaluation.

A further challenge of our approach was related to coding the active ingredients of the isolated intervention components. We felt it was important to use the same taxonomy to allow comparisons across intervention components. Therefore, we chose the BCTTv1 as this was the most widely used approach for coding behavioural interventions [47]. However, the taxonomy was more challenging to apply to the ACT component than others, and several ACT specific intervention methods could not be positioned in the BCTTv1. This highlighted that the BCTTv1 taxonomy does not comprehensively cover all techniques that are involved in ACT based interventions; a limitation also acknowledged elsewhere [156].

In using theory to develop the intervention components, we identified barriers to AET adherence to be targeted, and then considered psychological theories relevant to each barrier. This enabled us to consider theories specific to each identified determinant. An alternative approach could be to begin with a theory, and develop intervention components based on the constructs of that theory. However it has been recommended not to rely on singular theories when developing interventions to target medication adherence as single theories do not fully explain this behaviour [157]. Our approach enabled exploration of multiple theories to inform the development of our intervention components.

Using factorial trials to evaluate multiple intervention components, as suggested by the MOST framework, is a relatively new approach in health services research. We made adaptations to IM based on time available and to include important considerations guided by MOST [28, 31]. Strengths of our approach include applying an established intervention development method within the MOST framework, and the systematic reporting of the intervention development process. The differing formats of the intervention components allowed each determinant to be targeted using the most appropriate modality for that determinant. However, evaluating different formats of components may confound the mechanism of the intervention with the content. For example, participants may find the ACT component more engaging due to interaction with a therapist, rather than due to the content of the component. Future work could account for this by using a placebo control; for example by comparing ACT delivered by a therapist with an equivalent amount of time with a therapist discussing a different topic.

## Conclusions

We have developed a complex behavioural intervention package aiming to support AET adherence in women with breast cancer, made up of four intervention components. We have also demonstrated how IM can be harnessed to develop an intervention package that targets known determinants of medication taking behaviour in this population. Guided by MOST, this intervention package will be optimised in further trials with the aim of defining effective, efficient and scalable strategies to support behaviour change.

## Supplementary Information

**Additional file 1.** Table to display registered clinical trials of interventions to support adjuvant endocrine therapy in breast cancer patients.

**Additional file 2.** Intervention component examples. This provides examples of the four intervention components that were developed; SMS messages, information leaflet, ACT participant manuals and the side-effect management website.

**Additional file 3.** Behaviour change techniques present in intervention components.

**Additional file 4.** TIDieR checklist.

## Data Availability

Data sharing is not applicable to this article as no datasets were generated or analysed during the current study.

## Abbreviations

AET: Adjuvant endocrine therapy
MOST: Multiphase Optimisation Strategy
IM: Intervention Mapping
SMS: Short messaging service
ER+: Oestrogen receptor-positive
MRC: Medical Research Council
RCT: Randomised Control Trial
PPI: Patient and Public Involvement
BCT: Behaviour Change Technique
ACT: Acceptance and Commitment Therapy
CSM: Common-sense model of illness representations
NHS: National Health Service
IAPT: Improving Access to Psychological Therapies
