# Supplementary material for: Supporting adjuvant endocrine therapy adherence in women with breast cancer: the development of a complex behavioural intervention using Intervention Mapping guided by the Multiphase Optimisation Strategy
Source: BMC Health Serv Res. 2022 Aug 24;22:1081. doi: 10.1186/s12913-022-08243-4 (PMC9404670; doi:10.1186/s12913-022-08243-4)
Supplement: Supplementary file 1 — Additional file 1. Table to display registered clinical trials of interventions to support adjuvant endocrine therapy in breast cancer patients. [file 12913_2022_8243_MOESM1_ESM.docx]

Additional File 1. Registered clinical trials of interventions to support adjuvant endocrine therapy in breast cancer patients

| **Clinical Trial ID** | **Description of intervention** | **Intervention modality** | **Design** | **Population** | **Status** | **Adherence related outcomes** | |
| --- | --- | --- | --- | --- | --- | --- | --- |
| NCT03592771 | Web-enabled app in which patients input their treatment-related symptoms or changes. Reported symptoms integrate into electronic health care records. Concerning symptoms trigger an alert to the care team and contact is made.  App group: receive weekly reminders (via text or email) to use the app  App + Feedback group: receive weekly reminders and feedback about their use of the app | App and text/email reminders | 3 arm RCT; usual care vs app vs app plus feedback | AI or Tamoxifen | Recruiting | Electronic pillbox monitoring |  |
| NCT04142476 | Motivational, semi-directed interviews with pharmacists over 18 months, to motivate adherence to hormone therapy. | In person | No randomization; Single group assignment | Any AET | Recruiting | Data from electronic pillboxes |  |
| NCT04861896 | Smartphone app with a 12-week program regarding psychoeducation about breast cancer and hormone therapy, stress awareness and management, social support, and enhanced communication and intimacy skills. | App | No randomization; single group assignment | Hispanic/Latina women, any AET | Recruiting (for pilot trial) | Adherence to Refills and Medications Scale |  |
| NCT04824339 | 8 week aerobic and resistance program with virtual group based supervised exercise sessions twice per week (60 minutes). Optional information on healthy eating. | Virtual exercise sessions via Zoom | Randomized, partial crossover; immediate intervention vs delayed intervention | Tamoxifen or AI | Recruiting | Voils DOSE nonadherence measure (secondary outcome) |  |
| NCT04651452 | Values affirmation group: participants asked to write an essay monthly for 6 months about values important to them.  Reflective journal group: Participants will be asked to write monthly essays for 6 months about their daily routines, and values not important to them that could be important to others. | Online website or postal | RCT; value affirmation vs reflective journaling | AI | Recruiting | Morisky measure of adherence, and electronic pill bottle monitoring |  |
| NCT04719455 | HCP visits; baseline visit will include motivation, collaborative goal setting and plans for adherence and physical activity. Follow ups with HCP include personalized visual reports of medication intake, number of steps, and to identify any problems and solutions. | In person | Pilot RCT; usual care vs self-management intervention | Any AET | Recruiting (for pilot trial) | Number of days of missed medication (adherence is a secondary outcome) |  |
| NCT04176809 | One compulsory workshop about AET benefits. 2 optional workshops about nutrition and fatigue monthly. Monthly reminder letters sent including tips to deal with side effects. Regular HRQoL assessments using a tablet before consultations. | In person, and letters | RCT; standard care vs routine HRQoL assessment and therapeutic information | Any AET | Not yet recruiting | Morisky Green Levine scale |  |
| NCT04554927 | Web based application (no further information provided) | App | RCT; Web application vs active comparator (personalized schedule of medical follow up) | Any AET | Recruiting | Morisky 8 item adherence scale |  |
| NCT04086875 | Twice weekly SMS messages providing educational information for 6 months to motivate adherence | Text messaging | RCT; usual care vs text messages | Any AET | Recruiting | Smart pill bottles opening data |  |
| NCT02883361 | Motivational enhancement therapy. 4 in person counselling sessions over 12 months. Motivational interviewing to increase motivation and decrease ambivalence about change. | In person | RCT; Motivational interviewing vs attention control | AI | Not yet recruiting | Medication possession ration |  |
| CN-01810939 | Breast cancer information leaflet. Personalized letter to remind, motivate and inform patients about AET. Additional reminder phone calls from a study nurse. | Post, phone calls | 3 arm RCT; standard information vs personalized letters telephone calls | No information | No information | Self-report and prescription refill |  |
| NCT03949270 | Daily text messages asking whether the patient has taken their medication, Weekly messages asking about side effects. Monthly messages asking about barriers to adherence. Contact from physician if there are any concerning responses. | Text messaging | RCT; usual care vs text messaging | AI | Recruiting | Persistence to therapy at one year |  |
| NCT02707471 | Self-management intervention. 10 calls over 6 months delivered by a nurse, and tailored interactive voice messages based on adherence data. Focus on strategies for managing side effects, behavioural strategies to improve adherence and education. | Phone calls | RCT; self-management intervention vs general health education control | Any AET | Recruiting | Smart pill bottles (bottle opening and percent of pills remaining) |  |
| NCT02850939 | Interactive smartphone app that was personalized and culturally tailored. Additional support from a patient navigator. Focus on patient education, reporting side effects, delivery of self care advice, simplified communication between patient and oncology team. | App and patient navigation | RCT; usual care vs app and patient navigation | Any AET | Recruiting | Prescribing and refill records and self-report data via mobile app. |  |
| NCT03837496 | 6 weekly one hour sessions in small groups of 2-3; psychoeducation, problem solving barriers to adherence, cognitive behavioural skills, relaxation training, coping strategies for side effects, and mindfulness techniques. Two individual 15 minute semi-structured interview with therapist one and two months after the intervention to problem solve ongoing challenges with adherence. | Videoconferencing | RCT; STRIDE intervention vs medication monitoring control (pilot trial) | Any AET | Recruiting | MEMS caps, MARS-5 (adherence is secondary outcome due to pilot trial) |  |

Key: RCT= Randomised Control Trial; AI = aromatase inhibitor; AET = adjuvant endocrine therapy; DOSE = Domains of Subjective extent of nonadherence; HCP = Health care practitioner; HRQoL = Health related quality of life; STRIDE = Symptom-targeted randomized intervention for distress and adherence to adjuvant endocrine therapy; MEMS = Medication event monitoring system; MARS-5; Medication adherence report scale.

Note: Where there were multiple publications regarding an ongoing trial (e.g. study protocols and development papers), the trial is only displayed in the ongoing interventions table to avoid repetition.
