# Supplementary material for: Supporting adjuvant endocrine therapy adherence in women with breast cancer: the development of a complex behavioural intervention using Intervention Mapping guided by the Multiphase Optimisation Strategy
Source: BMC Health Serv Res. 2022 Aug 24;22:1081. doi: 10.1186/s12913-022-08243-4 (PMC9404670; doi:10.1186/s12913-022-08243-4)

**Additional File 2- Intervention Component Examples**

**SMS Message Examples**

Take your medication consistently at the same point of your everyday routine. Within a couple of weeks, it should start to feel like 'second nature' to you.

Try popping a pen and calendar next to where you take your medication, and tick when you've taken it - the tick will remind you if you've taken it already.

Do you use your phone alarm to get you up in the morning? You could trying setting it with a daily message to take your medication.

When you are down to your last weeks worth of your medication, try to make it a rule that you order your new prescription at the same time.

You can check the NHS app for when you can next order a repeat prescription. Pop a calendar entry in your phone to remind you when that is.

If you go away, it could be useful to take your medication out of your bag and put it somewhere that you will see it

**Information Leaflet Example Pages**

**
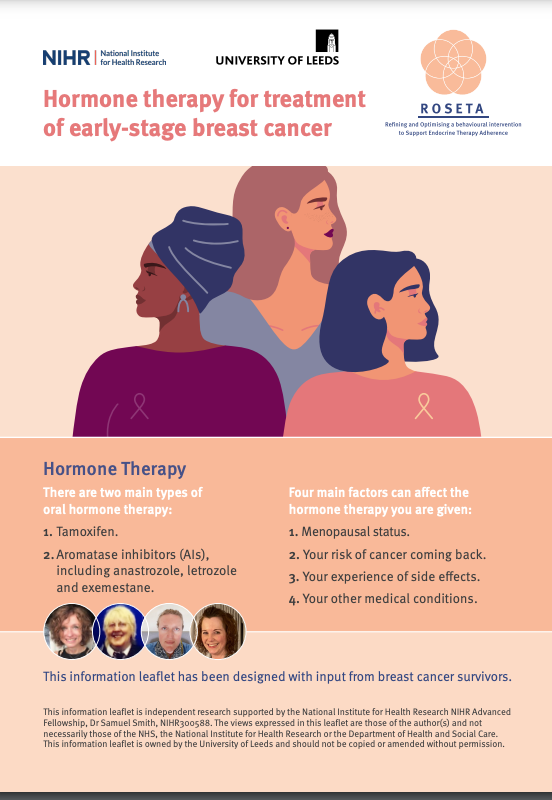
**

**
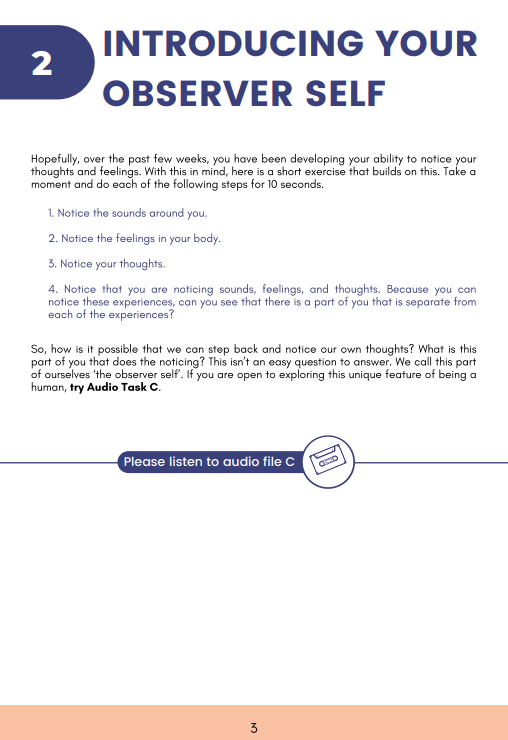

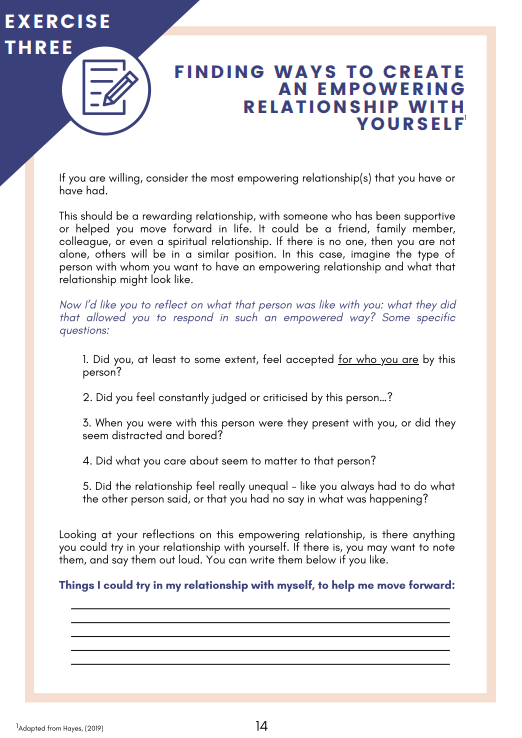

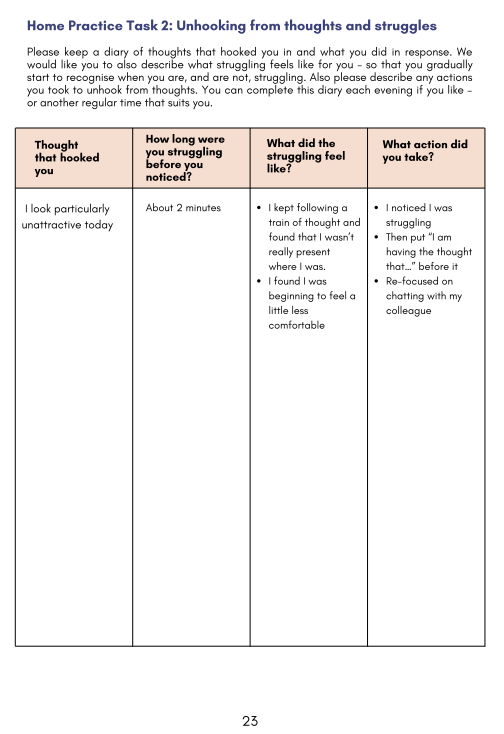

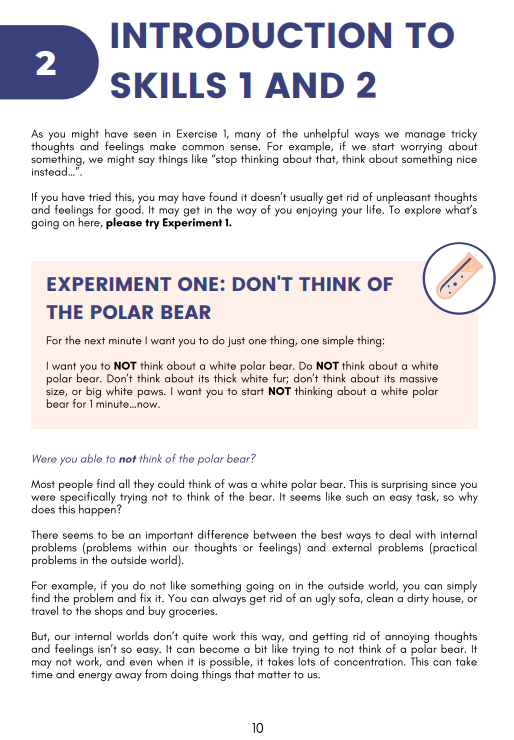
ACT Participant Manual Example Pages**

**Side-effect management website example pages**


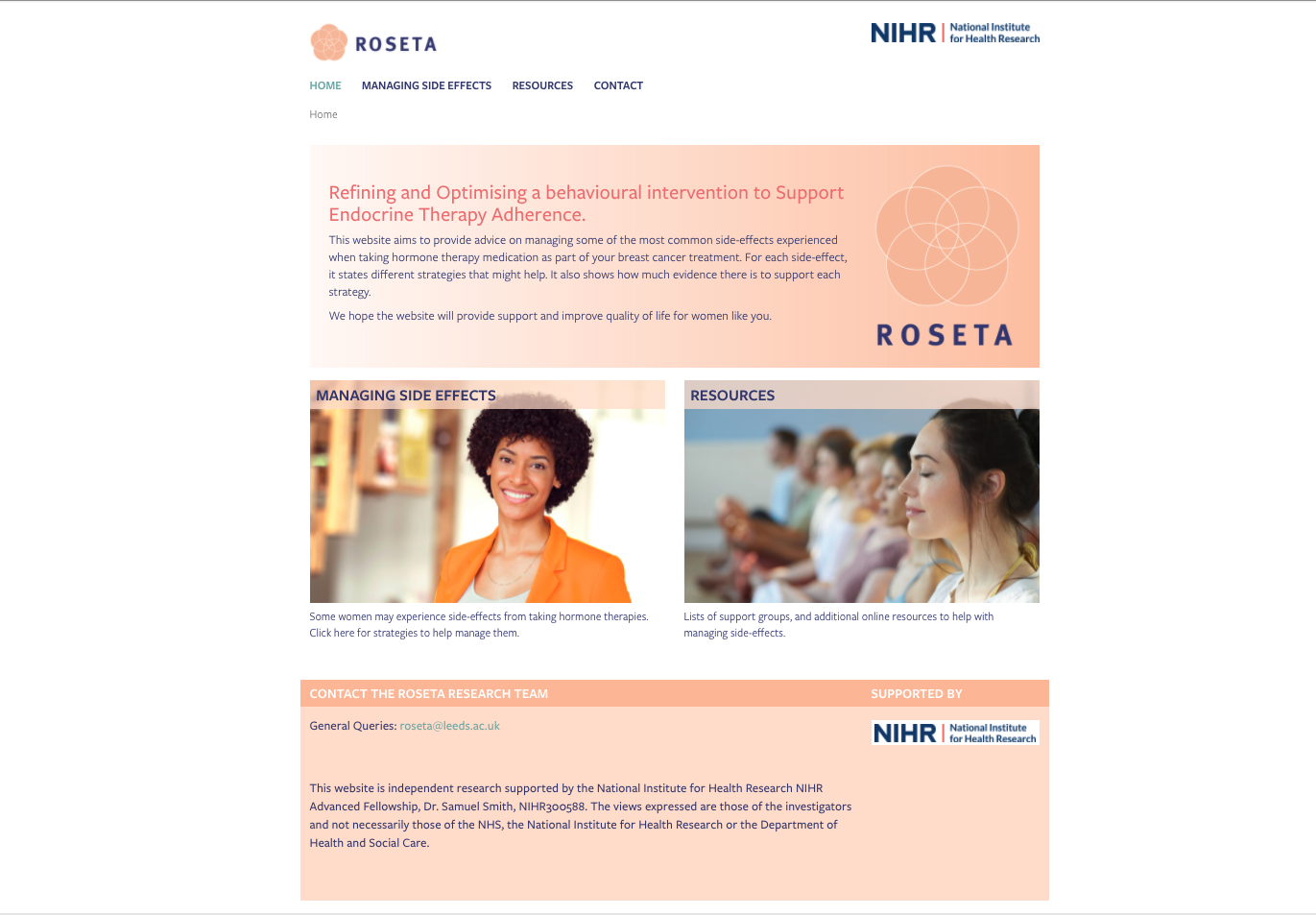


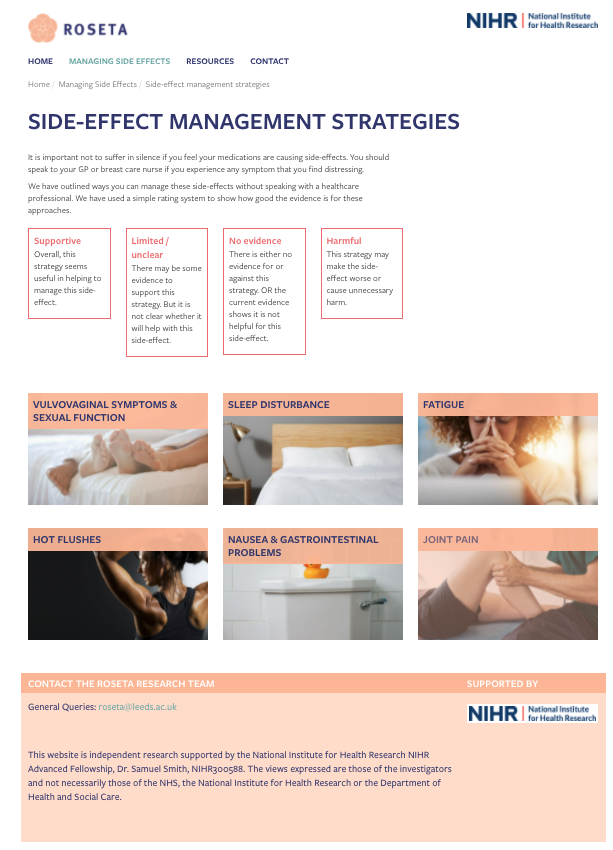


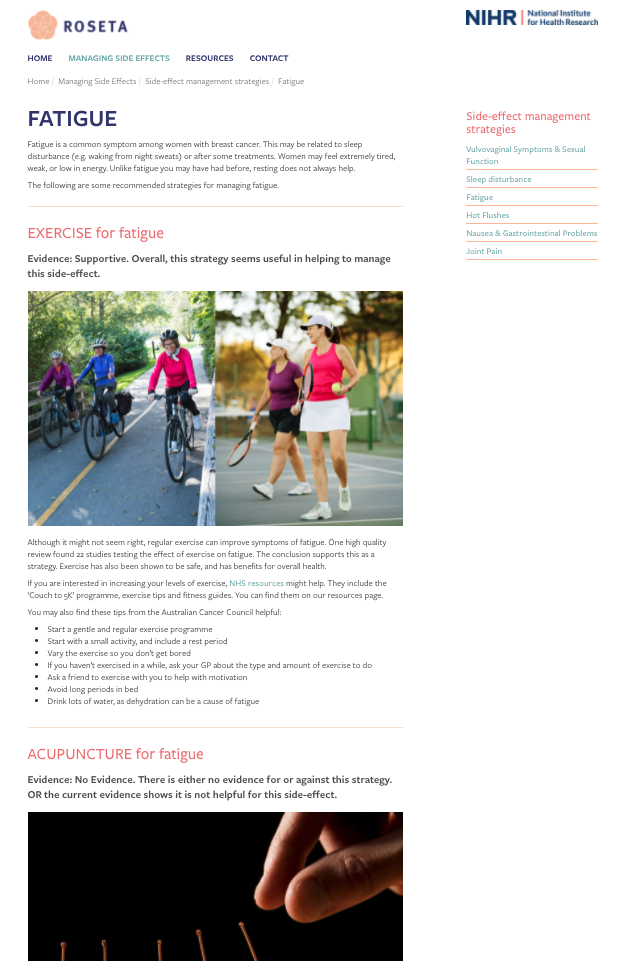

Supplement: Supplementary file 2 — Additional file 2. Intervention component examples. This provides examples of the four intervention components that were developed; SMS messages, information leaflet, ACT participant manuals and the side-effect management website. [file 12913_2022_8243_MOESM2_ESM.docx]
