# Supplementary figures and images for: Supporting adjuvant endocrine therapy adherence in women with breast cancer: the development of a complex behavioural intervention using Intervention Mapping guided by the Multiphase Optimisation Strategy
Source: BMC Health Serv Res. 2022 Aug 24;22:1081. doi: 10.1186/s12913-022-08243-4 (PMC9404670; doi:10.1186/s12913-022-08243-4)

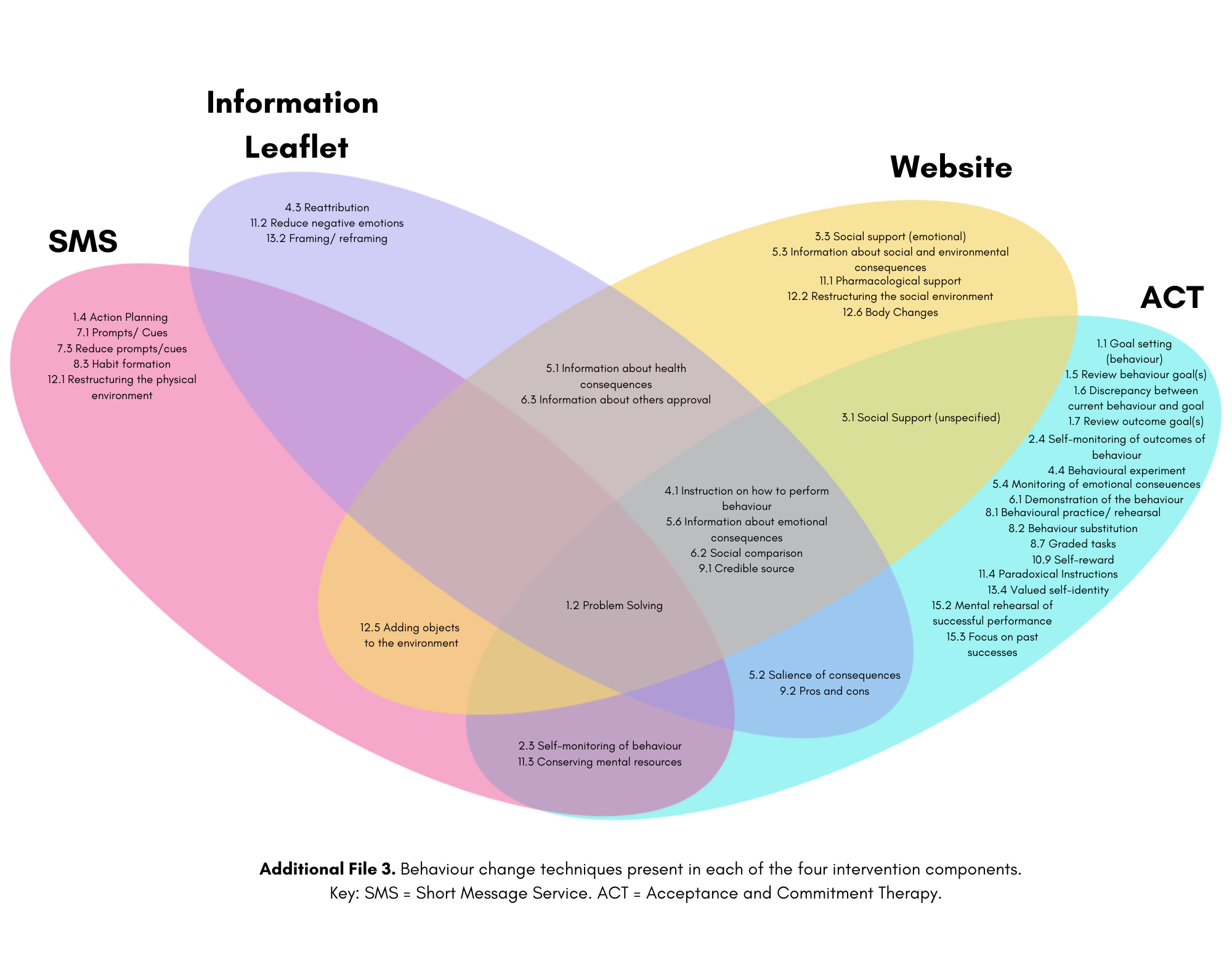

Supplement: Supplementary file 3 — Additional file 3. Behaviour change techniques present in intervention components. [file 12913_2022_8243_MOESM3_ESM.png]
