# Supplementary material for: Supporting adjuvant endocrine therapy adherence in women with breast cancer: the development of a complex behavioural intervention using Intervention Mapping guided by the Multiphase Optimisation Strategy
Source: BMC Health Serv Res. 2022 Aug 24;22:1081. doi: 10.1186/s12913-022-08243-4 (PMC9404670; doi:10.1186/s12913-022-08243-4)
Supplement: Supplementary file 4 — Additional file 4. TIDieR checklist. [file 12913_2022_8243_MOESM4_ESM.docx]

## The TIDieR (Template for Intervention Description and Replication) Checklist*:

| **N^o^** | **What** | **Details** |
| --- | --- | --- |
| **1** | **Name** | Refining and Optimising a behavioural intervention to Support Endocrine Therapy Adherence (ROSETA) |
| **2** | **Why: Rationale, theory, goal** | Adjuvant hormone therapies are prescribed at the end of hospital-based breast cancer treatment in order to prevent recurrence and mortality. However adherence to these medications is often poor, due to multiple factors, including forgetfulness, beliefs about medications, intolerable side effects and psychological distress. Previous adherence interventions have tended to consist of solely educational based interventions that are not grounded in theory, and did not target the factors commonly associated with medication adherence. An intervention targeting a range of factors that have been highlighted as barriers to adherence in needed.  *Memory and forgetting*  Mobile phone-based interventions are well suited to tackle forgetfulness as a barrier to adherence, through promotion of habit formation. SMS messages have been shown to be effective in improving medication adherence in other chronic illnesses but have not been widely tested in cancer patients.  *Medication schemas*  Accurate information about the necessity and risks of AET has the potential to increase women’s perceptions of their need for AET, and to reduce unfounded concerns about the medication. Women with breast cancer have stated that they would like more accurate information about AET to overcome unfounded concerns.  *Psychological Flexibility*  Acceptance and Commitment Therapy (ACT) has been shown to improve outcomes in those living with chronic illness, chronic pain, and cancer. ACT aims to increase a participant’s awareness of their personal values, and to undertake more of the behaviours that support these values – a process that often involves developing a willingness to have painful thoughts and feelings (such as medication side-effects). ACT targets psychological flexibility, which can improve functioning during objectively difficult circumstances, and can often reduce psychological distress as a by-product.  *Living with Side effects*  One of the most commonly cited barriers to AET adherence is the impact of side effects, and the lack of support for management of these is commonly cited. There are a number of strategies for these side effects that have the potential to be effective in alleviating symptoms. However, these are typically not presented in a patient-friendly manner.  Given the above, we have co-designed four intervention components for women with breast cancer who have been prescribed adjuvant endocrine therapies; SMS reminder messages to target forgetfulness, an information leaflet to promote necessity beliefs and reduce concerns, ACT therapy sessions to address psychological distress, and a side-effect management website to support living with side effects. The aim of the intervention components are to support medication adherence to hormone therapy. |
| **3** | **What Materials** | *SMS component*  This component involves 43 SMS messages being sent over four months. This includes three opening messages, one closing message, 36 messages related to behaviour change techniques aiming to promote habit formation, and 3 messages (sent monthly) as a reminder that participants can stop any further SMS messages being sent. The content of the SMS messages was co-developed with experts in behaviour change and/or medication adherence, and women who have experienced breast cancer.  *Information leaflet*  Participants will receive an information leaflet containing detailed information about AET. This includes information about how the medication works (with diagrams to supplement), information about the benefits and side effects of AET, answers to common concerns that women have, and quotes from women with experience of taking AET.  *ACT*  Participants will receive a participant manual consisting of information about the ACT skills and home practice tasks, in addition to corresponding audio files to assist with the home practice tasks. Each of the four modules focused on a different ACT-based skill:   - Module 1: Mindfulness and unhooking - Module 2: Following your values - Module 3: Taking an observer perspective - Module 4: Recap, reflection, and staying committed   *Therapists delivering ACT sessions*  Therapists delivering the intervention will receive two half days of bespoke training delivered by clinical psychologists with ACT experience. Alongside this, they will receive a training manual, with information about ACT generally, and specific session plans for the intervention sessions.  *Side-effect website*  Participants will receive access to a bespoke website containing information and strategies for self-management of side effects, and signposting to further sources of support. |
| **4** | **What Procedures** | ***Intervention Delivery***  *SMS component*  Participants will receive 43 SMS messages over four months. The 36 messages relating to behaviour change techniques will be sent on the following schedule:   - Daily messages for 2 weeks - Two messages per week for 8 weeks - Weekly messages for 6 weeks   *Information leaflet*  Participants will be emailed the information leaflet.  *ACT*   - 4x guided self-help modules consisting of information about ACT, home practice exercises and corresponding audio files - 1x 15-minute individual introductory session with a therapist - 3 x 25 minute individual support sessions with a therapist to discuss the module completed over the past week, their experiences of the home practice exercises, and to allow reflection on using the skills in their everyday lives. - 1x 15-minute closing session with a therapist   *Side-effect website*  Participants will be emailed log in details to access the website. |
| **5** | **Who provided** | *SMS messages*  SMS messages will be sent automatically by the Leeds Clinical Trials Research Unit (CTRU).  *Information Leaflet*  A research nurse will send the information leaflet via email.  *ACT*  The therapists who will deliver the intervention will receive two half days of training in delivering Acceptance and Commitment Therapy. The training will be delivered by Dr Chris Graham (CG), who has expertise in ACT applied to chronic disease. Training will include teaching about ACT and practice of intervention-specific therapy methods.  Each site’s therapists will have had a varied background that may or may not have included previous ACT training prior to our delivered training programme. However, all therapists will be Health and Care Professional Council (HCPC) registered practitioner psychologists (Clinical, Health or Counselling Psychologist).  *Side-effect website*  Access to the bespoke website will be emailed by a research nurse. |
| **6** | **How: mechanisms of delivery** | *SMS component*  SMS messages will be sent in an automated fashion by the CTRU to the participants mobile phone based on the following schedule:   - Daily messages for 2 weeks - Two messages per week for 8 weeks - Weekly messages for 6 weeks   *Information leaflet*  Participants will be emailed a copy of the information leaflet.  *ACT*  The individual sessions (5 in total) will be delivered via phone or video conferencing.  The participant manual containing information about each module, home practice tasks, and audio files will be emailed to each participant by the therapist following each session.  *Side-effect website*  Participants will be given a login to the website and will be able to use this as they wish. |
| **7** | **Where: location of delivery** | *SMS Messages*  Not applicable.  *Information Leaflet*  Not applicable.  *ACT*  All sessions will be delivered remotely via phone or videoconferencing.  *Website*  Not applicable |
| **8** | **When and how much** | *SMS component*  SMS messages will be sent by the CTRU based on the following schedule:   - Three opening messages - Daily messages for 2 weeks - Two messages per week for 8 weeks - Weekly messages for 6 weeks - One closing message - One message after 4, 8 and 12 weeks indicating the participant can stop the SMS messages at any time   *Information leaflet*  Participants will be emailed a copy of the information leaflet.  *ACT*  The introductory session will last 15 minutes, three subsequent sessions will last 25 minutes, and the final closing session will last 15 minutes. The therapy sessions will be held weekly.  *Side-effect website*  Participants will be given a login to the website and will be able to use this as they wish. |
| **9** | **Tailoring** | *SMS*  The same SMS messages will be sent in the same order to each participant.  *Information Leaflet*  The same information leaflet will be sent to each participant.  *ACT*  The deliverer may adapt the content to ensure it’s relevant to each participant (e.g. through discussing specific individuals’ values, goals, and behaviours).  *Website*  The website will be the same for each participant. |
| **10*** | **Modifications** | *<To be completed post study completion>* |
| **11** | **How well (planned)** | *SMS*  Successful delivery and receipt of the SMS messages will be recorded by the CTRU, alongside the number of SMS messages that were unable to be delivered. Participants will answer a single item asking whether they received the SMS messages, and another item asking how many of the SMS messages they read. Semi-structured interviews will be conducted to understand fidelity of receipt and enactment of the messages.  *Information Leaflet*  The number of information leaflets sent out to participants will be recorded. Participants will be asked to self-report whether they received the information leaflet, and how much of the information leaflet they read. Semi-structured interviews will be conducted with participants to understand the fidelity of receipt and enactment of the information leaflet.  *ACT*  Clinician fidelity to competently deliver the intervention in line with ACT will be assessed by an external rater with a background in ACT. They will complete the ACT-FM therapist stance subscale checklist whilst listening to the audio recording of 10% of sessions. A score of >4 on ACT consistent behaviours and <5 on ACT inconsistent behaviours is considered competent.  Additionally, an intervention specific metric of “Procedural Fidelity” will be completed, which measures other aspects of the intervention that are important for treatment fidelity but are not ACT-specific (e.g. reflecting on home practice tasks, sending module content etc). Therapists will complete the procedural fidelity checklist following each session. A percentage score is created for each session by dividing the score achieved by the maximum possible score achievable within that session and multiplying by 100.  Fidelity of ACT training will be monitored through Dr Graham assessing the recording of each therapists first ACT session, and rating competency based on the ACT-FM therapist stance subscale. Semi-structured interviews with the ACT therapists will assess the fidelity of training and delivery of the ACT component.  Participant fidelity to the ACT component will be monitored by recording the number of sessions attended, missed and cancelled. The therapist will additionally report how much of the module materials the participant has read and engaged with (participant manual, audio files and home practice tasks). Participants will be asked to self-report receipt of the module content, and engagement with the participant manual, audio files and home practice tasks. Semi-structured interviews will additionally assess fidelity of receipt and enactment.  *Side-effect website*  Website data will be tracked for each participant, including number of logins, time spent on pages, videos watched and clicked links. Participants will be asked a single item about their engagement with the website. Fidelity of receipt and enactment will be additionally assessed through semi-structured interviews. |
| **12*** | **How well (actual)** | *<To be completed post study>* |
